# Dietary lactoferrin supplementation to gilts during gestation and lactation improves pig production and immunity

**DOI:** 10.1371/journal.pone.0185817

**Published:** 2017-10-12

**Authors:** Marefa Jahan, Susie Kracht, Yen Ho, Ziaul Haque, Birendra N. Bhattachatyya, Peter C. Wynn, Bing Wang

**Affiliations:** Graham Centre for Agricultural Innovation, Charles Sturt University and NSW Department of Primary Industries, Wagga Wagga, NSW, Australia; Xavier Bichat Medical School, INSERM-CNRS - Université Paris Diderot, FRANCE

## Abstract

Lactoferrin (LF), a sialylated iron-binding glycoprotein, performs multiple beneficial functions including modulating immunity and improves neurodevelopment, health and growth performance. Maternal LF intervention for gilts (first parity sows) on the performance of gilts and their offspring remains unknown. In the current study gilts were fed with a commercial pig feed supplemented with 1g LF /day (treatment group) or 1g milk casein/day (control group) from day 1 post mating throughout pregnancy and lactation for about 135 days. The milk production and body weight gain was monitored. The immunoglobulin concentrations in the serum of gilts and piglets were measured using ELISA. Our study showed that maternal LF supplementation to the gilt (1) significantly increased milk production at different time points (day 1, 3, 7 and 19) of lactation compared to the control (p<0.001); (2) significantly increased body weight gain of their piglets during the first 19 days of life compared to the control group (p<0.05); (3) tended to increase pregnancy rate, litter size and birth weight, number of piglets born alive, and decrease the number of dead and intrauterine growth restriction (IUGR) piglets; (4) significantly increased the concentration of serum IgA in gilt and serum sIgA in piglet (p<0.05). In summary, maternal Lf intervention in gilts can improve milk production, pig production and serum IgA and sIgA levels, and therefore plays a key role in shaping the performance of their progeny.

## Introduction

Lactoferrin (LF) is a 80 kD non-haem iron-binding glycoprotein that is part of the transferrin protein family [1, 2] and consists of ca 703 amino acids with high homology among species. Multiple sialic acid (Sia) residues are attached to the N- linked glycan chains and the polypeptide chain is folded into 2 lobes [3]. LF is first expressed at the two- to four-cell stage of embryonic development and continues until the blastocyst stage of pre-implantation. Expression of LF is again resumed in the latter half of gestation, where it is detected in neutrophils and in epithelial cells of the developing digestive and respiratory tracts [4]. In adult mammalian species LF is produced by mucosal epithelial cells. It is found in various mucosal secretions, including tears, saliva, vaginal fluids, semen [5], nasal and bronchial secretions, bile, gastrointestinal fluids and urine [6]. However the highest concentrations of LF are detected in colostrum (~9.7 g/L) and mature milk (2–3 g/L) of humans, making it the second most abundant whey protein in human milk [7]. Bovine mature milk contains approximately one tenth the amount of LF as human milk, ranging from 0.03–0.1 g/L [8]. In sow milk however, LF concentration in colostrum is about 1359 μg/ml and in mature milk is about 408–924 μg/ml [9]. LF is also highly expressed in secondary neutrophil granules (15μg/10^6^neutrophils) [10] and in bodily fluids such as blood plasma and amniotic fluid. In addition to constitutive expression at the mucosal surface, LF is differentially regulated by hormones and transcription factors in a tissue-specific manner [2]. For instance, in the mammary gland LF expression is under the control of prolactin, whereas in the reproductive tract, the expression of this protein can be induced by the steroid hormone estrogen [11].

LF has several important physiological functions and most research has focused on LF action as a modulator of immune function and its involvement in the host defence response against a spectrum of bacteria (Gram+ and Gram−), fungi, yeasts, viruses [12] and parasites [13], as well as stimulating the growth of probiotic bacteria such as *Lactobacillus* and Bifidobacteria [14]. It also participates in intestinal iron homeostasis, promotes bone growth, and inhibits the growth of some human cancers [15–17]. LF up-regulates intestinal gene expression of brain-derived neurotrophic factors (BDNF), ubiquitin carboxy-terminal hydrolase L1 (UCHL1) and alkaline phosphatase activity to alleviate early weaning diarrhea [7] and promotes early neurodevelopment and cognition by upregulating the BDNF signaling pathway and polysialylation in postnatal piglets [18]. In human clinical studies, LF plays a protective role in reducing the incidence of invasive fungal infections in low birth weight infants [19] and late-onset sepsis in pre-term neonates [20]. LF can also prevent the development of necrotizing enterocolitis in very low birth weight neonates [21]. Its role in controlling immunity extends to the modulation of inflammation by a dose-dependent inhibition of langerhans cell migration and the accumulation of dendritic cells within the host’s lymph nodes [22]. Furthermore, LF is capable of inhibiting the pro-inflammatory activity of cytokines including interleukin 1β and IL-2 [22].

Intrauterine growth restriction (IUGR) refers to a condition of the mammalian embryo/fetus in which it does not reach its growth potential during pregnancy. In both humans and animals, IUGR neonates are reported to have higher perinatal mortality and morbidity associated with low efficiency of food utilization, and permanent stunting effects on postnatal growth and development [23], thus it presents a major problem for human medicine [24] and animal production [23]. The causes of IUGR in pig includes the first parity, a large litter size (>12), inadequate nutrition in utero, diseases, environmental stress, or the dysfunction of the placenta, endometrium, or uterus [24] associated with some physiological and production-imposed conditions in livestock [25–28]. In pigs, 15–25% of newborns weigh are 1.1 kg or less compared with a normal birth weight of 1.4 kg [23, 25, 29], thus IUGR is a greater problem in pigs than in any other domestic mammals [23]. The problems, such as pre-weaning mortality, reduced growth performance, preterm births and stillbirths, as well as, a high prevalence of infections are also common within the swine industry, and in particular with primiparous gilts [30, 31].

We hypothesized that LF have a positive effect on improving pig production. The objectives of the current study were to investigate the role of maternal LF supplementation during pregnancy and lactation in promoting milk production of gilts, growth and development of piglets, reducing the incidence of IUGR and still births.

## Materials and methods

### Animals and diet

Sixty healthy domestic gilts (*Sus scrofa*), aged ~220 days and body weight of 151.8 kg±15.1 kg, were randomly selected from five different breed lines at the Pig Improvement Company (PIC) Grong Grong Farm, New South Wales (NSW), Australia. These included breed line 2 which were pure bred Large Whites, breed line 3 which were pure bred Landrace and breed lines 4, 7 and 9 which were pure bred Duroc pigs. The gilts were inseminated three times in a two-day period. From day 1 of mating, the gilts were randomly assigned to treatment (n = 30) and control (n = 30) groups ensuring the same proportion of each breed line was represented in each group (S1 and S2 Tables). Nutritional intervention involved top-dressing of 1 g/d of LF (Tatura Milk Industries Limited) for treatment gilts and 1 g/d of casein (Murray Goulburn Co-Operative Co. Limited) for control gilts from day 1 post mating throughout gestation and then until the end of a 21d lactation period (total ~135 days). Pigs were fed a commercial gestation diet (S3 Table) of 2.4 kg/day from mating to the end of gestation, and then a lactation diet (S3 Table) of 5.8 kg/day over lactation until weaning. All animals were provided with water *ad libitum* and housed in individual stalls once mated throughout the experimental period. On day 30 post mating, the gilts were subjected to ultrasound (Draminski ANIMAL profi, Poland) to determine pregnancy. Any gilt confirmed non-pregnant was removed from the trial. At four days prior to farrowing (gestation length was 114±1 days), all pregnant gilts were transferred from the gestation shed to the farrowing shed in individual farrowing crates. Farrowing sheds were maintained at a constant temperature of 27°C. The study was approved by the Animal Care and Ethics Committee of Charles Sturt University, Wagga Wagga, NSW, Australia.

### Monitoring gilt live weight gain and fecal sample collection

A baseline live weight of gilt was recorded on the first day of mating. Then, gilt live weight was recorded on days 30, 60, 74, 90 and 104 post mating using Veterinary Scales (Slater Brecknell VD-1000 Vet Deck). Fecal samples were collected within 105–110 days of gestation from each gilt and stored at -80°C pending analysis.

### Monitoring pregnancy rate

There were three stages at which pregnancy rate of the treatment and control groups was monitored. The initial pregnancy rate was determined 30 days after the first mating using ultrasound. To determine the second pregnancy rate, the gilts that were confirmed negative from the first pregnancy test and returned to estrus, were mated again and the percentage of gilts testing positive represented the second pregnancy rate. The third pregnancy rate was generated from the weaned gilts in each group that were mated again approximately after 134 days from the commencement of the study.

### Monitoring piglets

On the day of farrowing, the total numbers of piglets born, and those born alive, dead and IUGR were recorded. The weight of the whole live litter at birth, individual weights of piglets at birth and at postnatal day 19 was recorded using a digital weighing scale. (Slater Brecknell ElectroSamson Digital Hand Held Scale, USA). All piglets were graded based on individual birth live weight into either >1000 g, 1000–900 g, 900–800 g, 800–700 g and < 700 g to determine the incidence of IUGR piglets.

In commercial farming systems, fostering piglets from one gilt/sow to another is an established swine husbandry practice. The purpose of this cross fostering is to reduce within-litter weight variation among piglets and to match more evenly the number of piglets with the dam’s ability to nurse them. In our study, the farm administration started cross fostering to ensure nursing of 10–11 piglets by each gilt after we recorded piglet ID and birth weight. Some of the piglets from our treatment and control group were cross-fostered to gilts/sows not enrolled in our study, while some piglets from non-experimental gilts/sows were fostered within our experimental groups. Only those piglets, which remained within the respective treatment (n = 114) or control (n = 80) group till weaning were weighed to record the weaning weight. Similarly, any piglets that were cross fostered into our experimental groups were excluded from recording the weaning weight.

### Measuring milk production of the gilts

Milk production of the gilts was measured at postnatal day 1, 3, 7 and 19 of lactation respectively. On each day, the piglets were separated from the gilt using a partition board for 60 minutes. After 60 minutes, the piglets were weighed using the digital weighing scales (Slater Brecknell ElectroSamson Digital Hand Held Scale) and then were allowed to suckle their dam by removing the partition. When it was confirmed that milk let down had occurred and the piglets had suckled for about 15 minutes, they were weighed again using the same scale and the weight gain of the litter was used as a direct indication of gilt milk production per suckling episode (weigh-suckle-weigh method).

Weighing of piglets post suckling was completed promptly to decrease the incidence of urination and defecation, which was recorded. But any urination or defecation of piglets whilst suckling or weighing was taken into account and the process was repeated in such cases when determining milk production. Thus, the majority procedure of weighing post suckling piglets was repeated at least for 4 times to confirm no defecation or urination by piglets occurred through calculating the mean of two weigh-suckle-weigh repetitions. Milk production per suckling episode was determined twice at each time point to allow accurate determination of gilt milk production. However, before postnatal day 19, 5 gilts from LF group and 7 gilts from control group failed to lactate satisfactorily and thus were eliminated from the shed with their litters before determined 19 days milk production (S4 Table). The causes of these cases of agalactia were not diagnosed.

### Immunoglobulin assay

Blood samples (2 ml) were collected from treatment and control gilt and 3 piglets selected randomly from each litter at day 19 of lactation via the jugular vein. Each blood sample was collected in a serum clot activator tube (Greiner bio-one, Australia) and centrifuged at 3000g for 10 minutes at 4°C. The serum samples were collected and stored at -80^0^ C pending analysis. Fecal sample preparation for secretory immunoglobulin A (sIgA) analysis was carried out following the instruction of the ELISA Kit (My Biosource, USA, Catalog no CSB-E12063p). Briefly, 100 mg of fecal sample was mixed with 5 ml of wash buffer on a vortex mixer until the mixture was homogenous. One ml of the mixture was transferred into an Eppendorf tube and was centrifuged for 10 min at 10000xg. The supernatant was diluted at 1:250 with wash buffer (4 μl + 996 μl wash buffer). 100 μl of the dilution was used in the test per well.

Pig immunoglobulin M (Ig M) ELISA Kit (Life Diagnostics, Inc. USA, Catalog no 5015–9), Immunoglobulin G (IgG) and Immunoglobulin A (IgA) ELISA Kit (Bethyl Laboratories, Inc. USA, Catalog no E101-104 and Catalog no E100-102) and pig sIgA ELISA kit were used to determine the blood serum IgM, IgG and IgA concentrations respectively. Pig sIgA kits were used to detect sIgA concentration in blood serum samples for both gilts and piglets as well as faecal samples of gilts only. All samples were analysed in duplicate. An automated plate washer **(**EL*x*50 Washer, BioTek) was used to wash the plates. A microplate spectrophotometer (SpectraMax, Bio-strategy) was used to measure the optical density of serum samples at a wave length of 450 nm. Only those serum samples, which were devoid of any haemolysis were used for analysis. The intra and inter coefficient of variation for the assay was less than 5%.

### Statistical analysis

Generalized linear mixed models (and binomial generalized mixed models where appropriate) were used to evaluate the effect of LF on gilt weight gain during gestation, milk production, piglet growth and development and immunity during lactation. Breed line was used as the random effect and group (or group and days where appropriate) were used as fixed effects. Residual plots were used to ensure that the model assumptions were met for all models. Differences in incidence of IUGR piglets between groups were compared using the Kaplan-Meier survival analysis. The differences between LF and control groups were considered significant at p<0.05. Data are presented as means ±SEM. The analysis was conducted using GenStat statistical software (17^th^ Edition).

## Results

### Live weight gain of gilts

**Fig 1:**
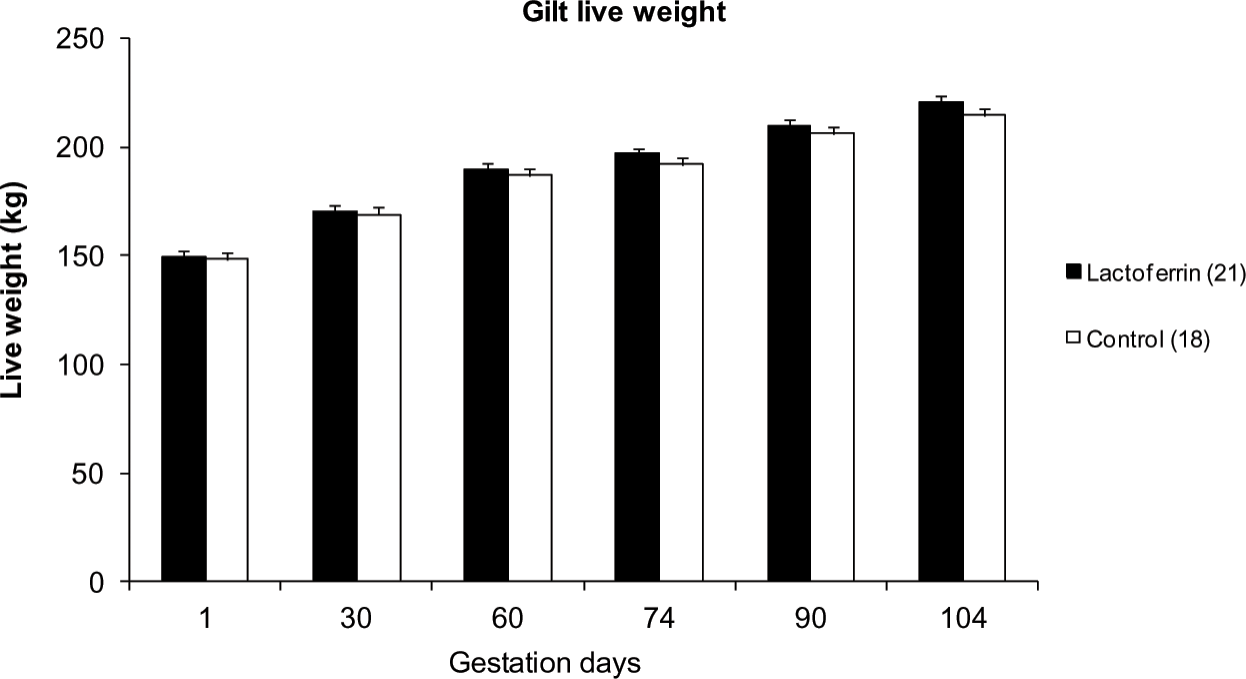
The effect of maternal oral lactoferrin supplementation (1g/day) during pregnancy (~114 days) on gilt live weight gain. Mean live weight of gilts during days1, 30, 60, 74 and 104 of pregnancy between treatment (LF 1g/day) and control (casein 1g/day). Values are mean ± SEM.

The mean live weight gain of gilts during gestation is shown in Fig 1. There was no significant difference in gilt live weight gain between the treatment and control groups (p>0.05), although live weights recorded on days 74, 90 and 104 of gestation were ~2–3% higher in the treated than control gilts at the same time point.

### Pregnancy rate

The pregnancy rate of gilts from the treatment and control groups for three different stages is summarised in Table 1. Out of 30 animals from each group 21 gilts from the treatment`group (73%) and 18 gilts from the control group (60%) became pregnant after the first mating. The non-pregnant gilts (9 from the treatment group and 12 from the control group) were subjected to a second mating after oestrus and a further 6 treatment gilts (69%) and 7 control gilts (58%) became pregnant. The same trend for a higher pregnancy rate was also observed when the weaned first parity sows were re-mated. Pregnancy rate was 86% (18/21) and 78% (14/18) for the treatment and control groups became pregnant respectively. However, no significance difference in pregnancy rates was found between the two groups (p>0.05).

**Table 1:** Incidence of pregnancy in lactoferrin (1g/day) supplemented and control groups.

	Lactoferrin group			Control group		
Mating time point	Total number	Success (no.)	Pregnancy rate	Total number	Success (no.)	Pregnancy rate
1^st^ mating	30	21	73%	30	18	60%
2^nd^ mating (returned gilts)	9	6	67%	12	7	58%
3^rd^ mating (weaned sows)	21	18	86%	18	14	78%

### Production related to litter

The mean number of piglets in litters of treatment and control gilts is shown in Table 2. There was no difference (p>0.05) in the litter size, nor in the number of born alive, stillborn, and total dead piglets between the maternal LF treatment and control groups (Table 2). Maternal LF supplementation tended (P = 0.09) to reduce the incidence if IUGR in piglets. We could not record the actual number of piglets at weaning for each treatment and control gilt, because of the need for cross fostering. However, we recorded the number of treatment and control piglets which remained with their dam until weaning (S1 Table).

**Table 2:** The effect of lactoferrin supplementation (1g/day) to the gilts during pregnancy and lactation (135 days) on the prolificacy of gilts and the fate of their piglets at farrowing.

Item		Lactoferrin Group (n = 21)	Control Group (n = 18)	P value
Litter Size		11.14 ± 3.58	10.22 ± 3.34	0.37
Alive piglets		10.14 ± 0.69	9.13 ± 0.75	0.26
IUGR piglets		1.14 ± 0.23	1.75 ± 0.33	0.09
Dead piglets		1.00 ± 0.21	1.25 ± 0.28	0.47
Total newborn body weight for whole litter (Kg)		14.38 ± 1.35	12.76 ± 1.47	0.16
Mean piglet body weight (kg)		1.41 ± 0.05	1.24 ± 0.07	0.08

Values are Mean ± SEM.

IUGR: Intrauterine growth retardation

### Incidence of IUGR

**Fig 2:**
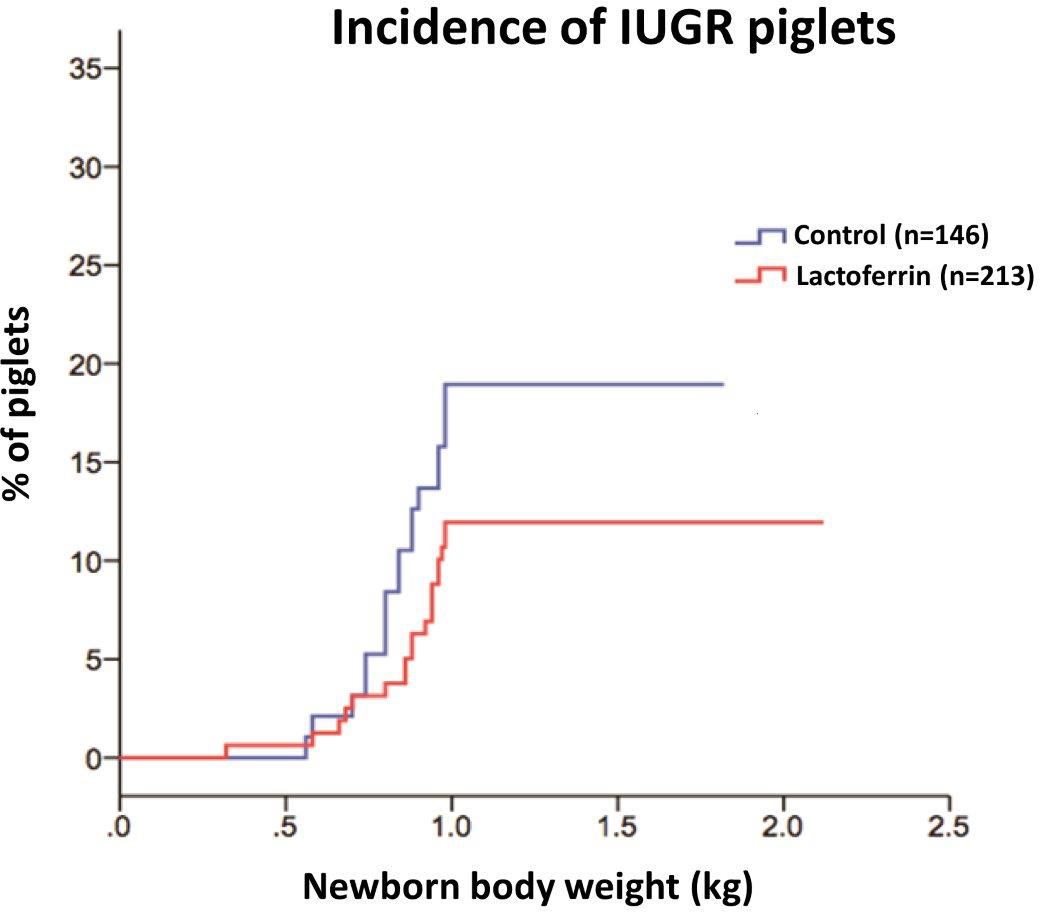
The effect of maternal lactoferrin (1g/day) supplementation during pregnancy and lactation (~135 days) on the incidence of intrauterine growth restricted piglets. n = 213 in LF group, n = 146 in control group (n = total number of alive piglets).

LF supplementation during pregnancy and lactation in gilts tended to reduce (p = 0.09) the incidence of IUGR in piglets. Around 12% of the alive newborn piglets in the LF group and ~ 20% in the control group were IUGR piglets (Fig 2).

### Early postnatal growth of piglets until weaning

**Fig 3:**
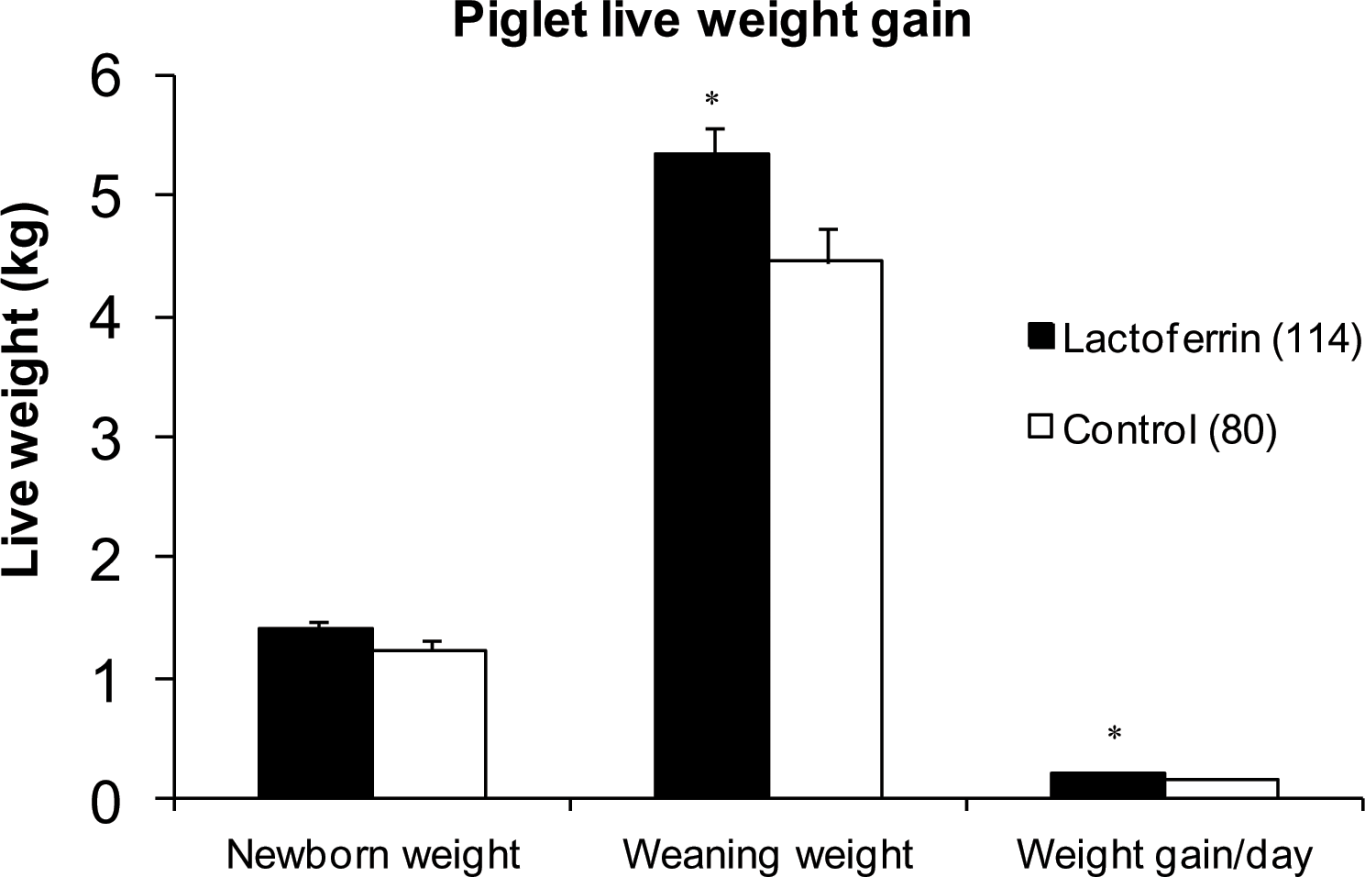
The effect of maternal lactoferrin supplementation during pregnancy and lactation (total ~ 135 days@1g LF /day) on piglet live weight gain. Newborn live weight, weaning weight and weight gain of piglets per day, *p<0.05. Values are mean ± SEM. (n = 114 in LF group, n = 80 in control group). Only those piglets which were retained with treatment or control gilts from birth to weaning were included.

The birth weight, weaning weight and live weight gain of piglets from day 1 to weaning (day 19) are presented in Fig 3. There was no significant difference in body weight of newborn piglets between LF treated (1.41±0.06 kg) and control (1.24±0.08 kg) groups, however piglets from LF treated gilts were significantly (p = 0.02) heavier (5.34±0.21 kg) at weaning compared with the control group (4.46±0.27 kg). This translated into a significant improvement in mean live weight gain per day from day 1 to day 19 in the LF treatment group (0.21 ± 0.01 kg) compared to the control group (0.17 ± 0.01 kg) (p<0.05, Fig 3).

### Milk production

**Fig 4:**
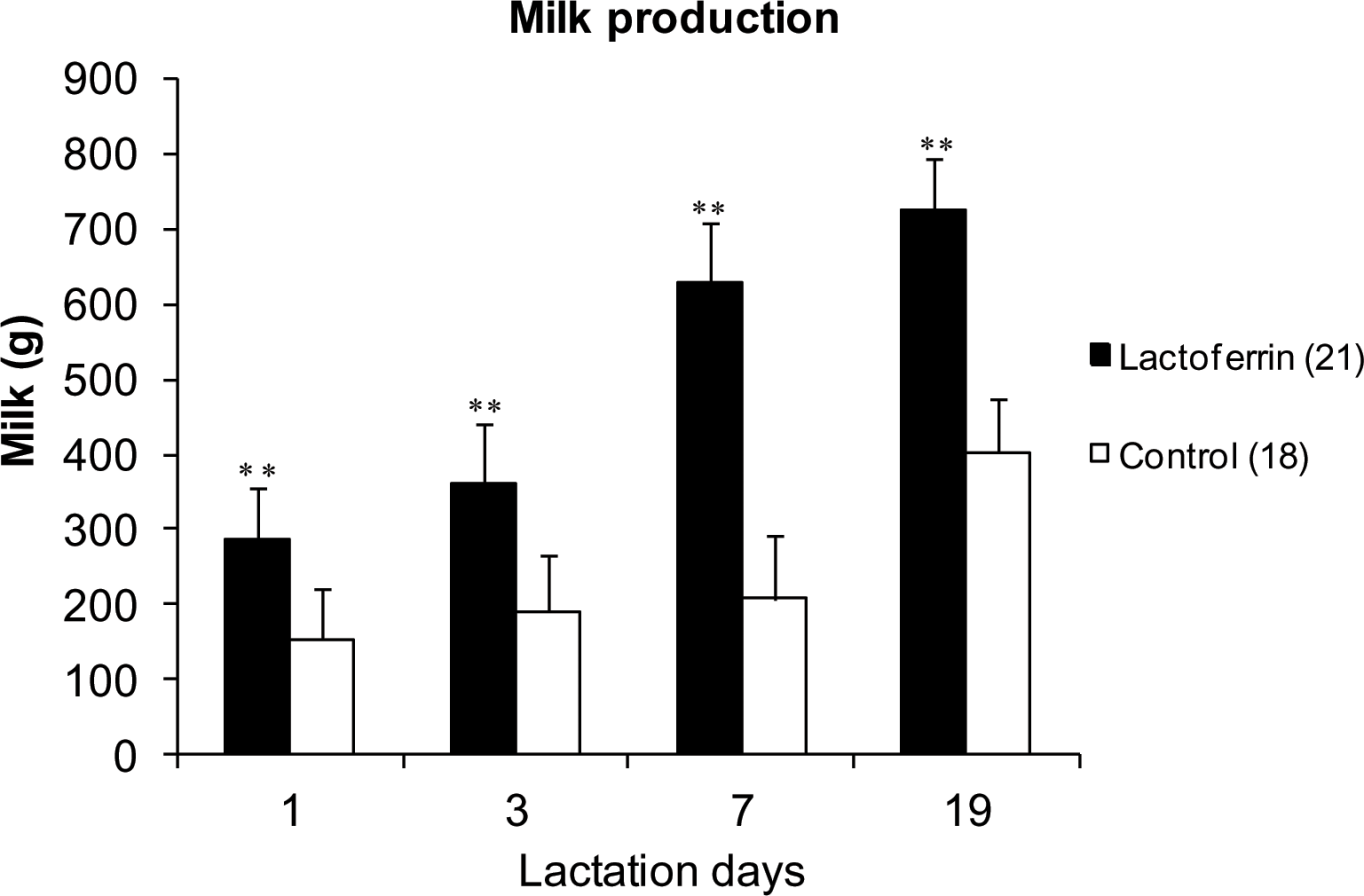
The effect of maternal lactoferrin supplementation (1g/day) during pregnancy and lactation (~135 days) on milk production of the gilts. Milk production by the gilts of treatment and control groups during days 1, 3, 7 and 19 of lactation, ** p < 0.001. Values are mean ± SEM. n = 21 in LF group, n = 18 in control group for day 1, 3 and 7 and n = 16 in LF group and n = 11 in control group for day 19.

Maternal Lf supplementation in gilts throughout pregnancy and lactation significantly increased milk production compared with the control at four time points of lactation (p <0.001, Fig 4). In the present study, after day 1, the cross fostering of piglets to equalize litter sizes across treatment and control group (n = 9.375 piglets in the LF group, and n = 9.309 in the control group) to ensure the suckling stimulus in terms of piglet numbers was the same in each gilt during milk production measurement on days 3, 7 and 19 of lactation. The measurement of milk production on day 1 were compromised by the uneven litter sizes (S4 Table).

### Serum immunoglobulin concentrations in gilts

**Fig 5:**
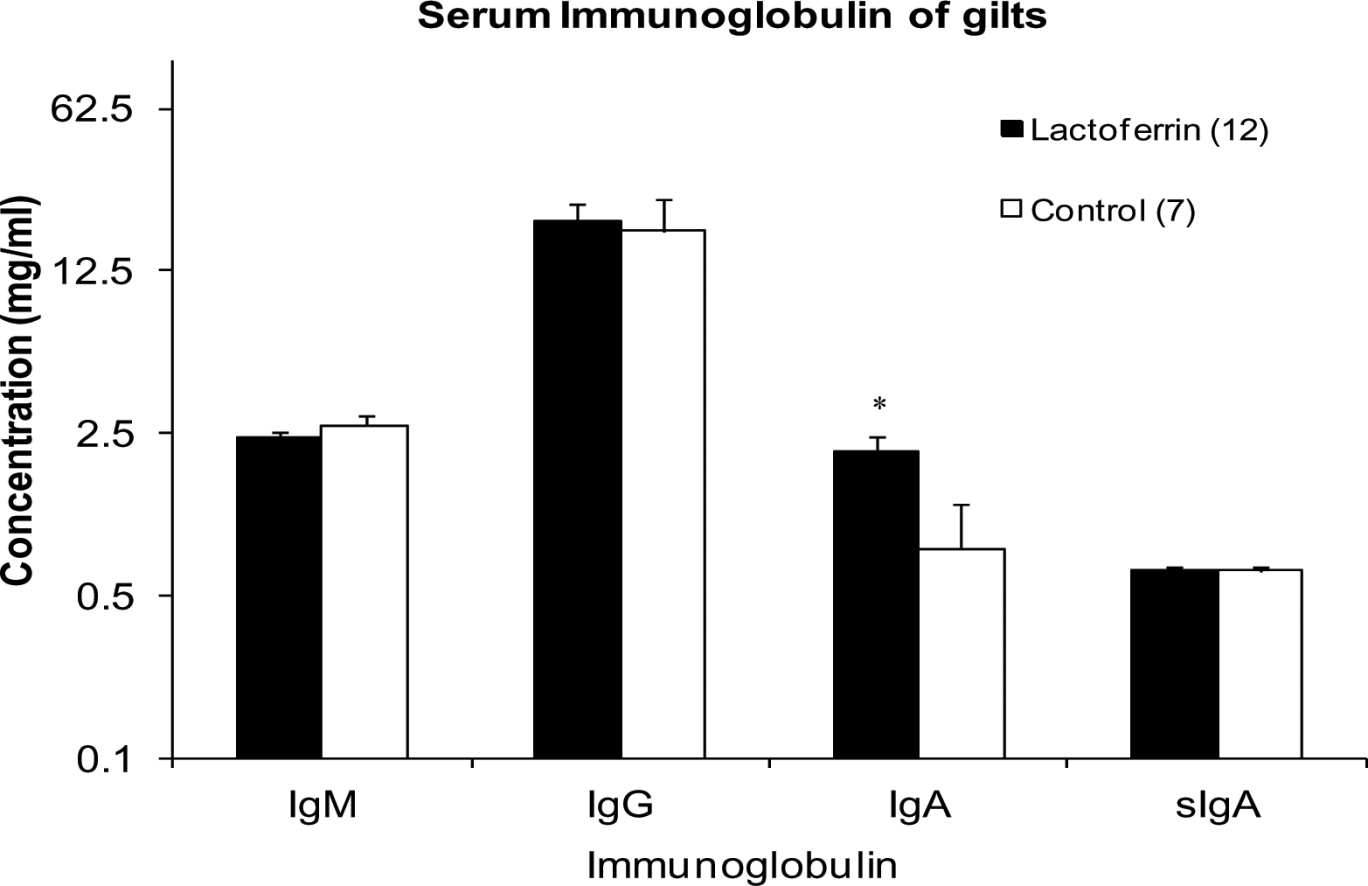
The effect of maternal lactoferrin supplementation (1g/day) during pregnancy and lactation (~135 days) on serum immunoglobulin concentration of gilts. Concentration of Immunoglobulin IgM, IgG, IgA and sIgA in serum samples of gilts collected before weaning on day 19 of lactation. *p<0.05. Values are mean ± SEM.

Mean concentrations of four different classes of circulating immunoglobulin in the serum collected from gilts at weaning are shown in Fig 5. The concentrations of IgM, IgG and sIgA were not significantly different between the treatment and control groups (p>0.05). However, a significantly higher concentration of IgA was found in the LF treated gilts (2.11 ± 0.33 mg/ml) compared to the control group (0.8 ± 0.45 mg/ml; p = 0.031).

### sIgA concentration in the faecal sample of gilts

**Fig 6:**
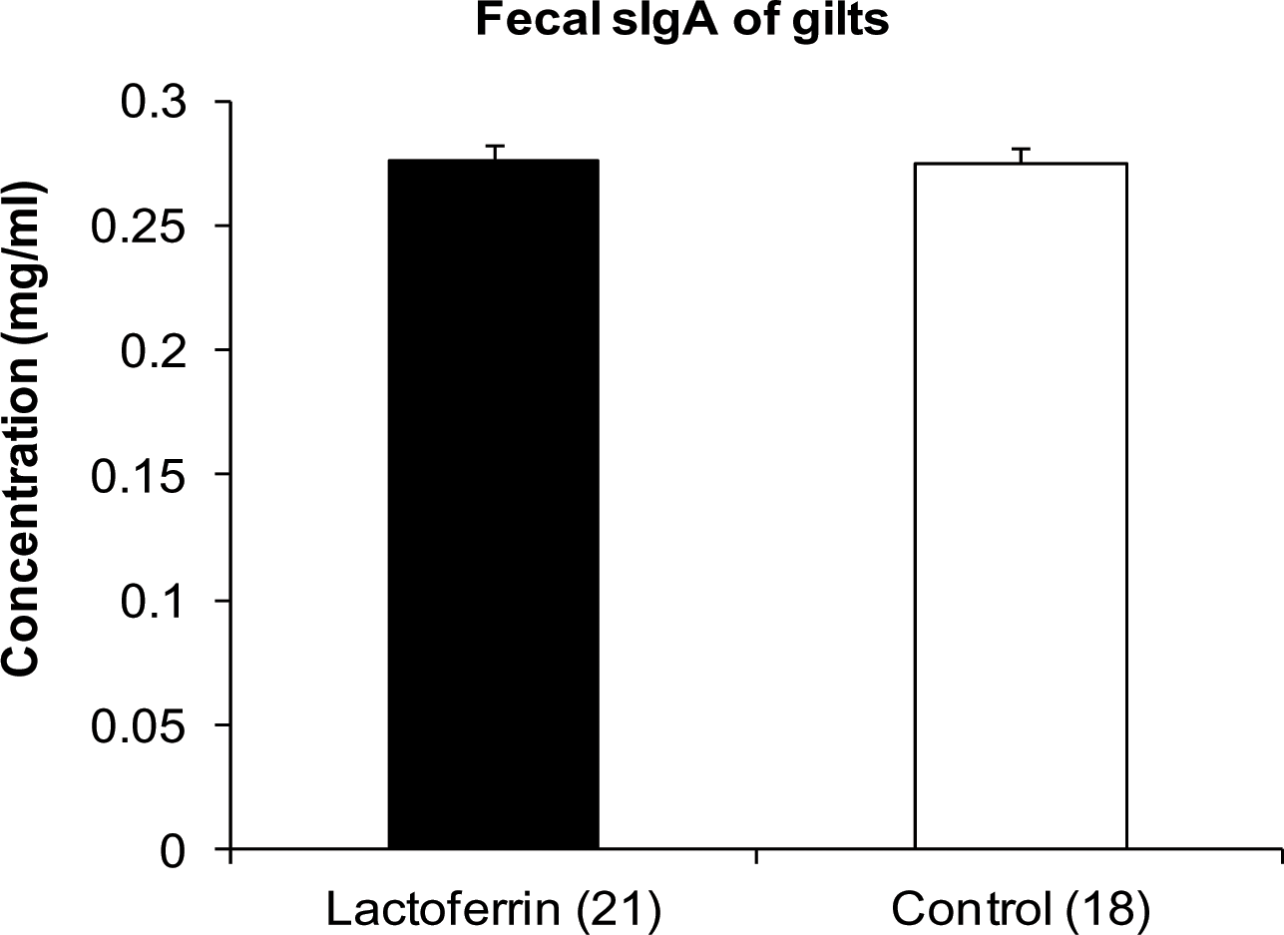
The effect of maternal lactoferrin supplementation (1g/day) during pregnancy and lactation (~135 days) on fecal sIgA concentration of gilts. Concentration of Immunoglobulin sIgA in the fecal sample of gilts collected before farrowing. Values are mean ± SEM.

The mean sIgA concentration in the fecal sample of gilts collected during the last week of gestation is showed in Fig 6. The concentrations of fecal sIgA for the LF treatment and control group was the same as 0.27 ± 0.006 mg/ml.

### Immunoglobulin concentration in the serum of piglets

**Fig 7:**
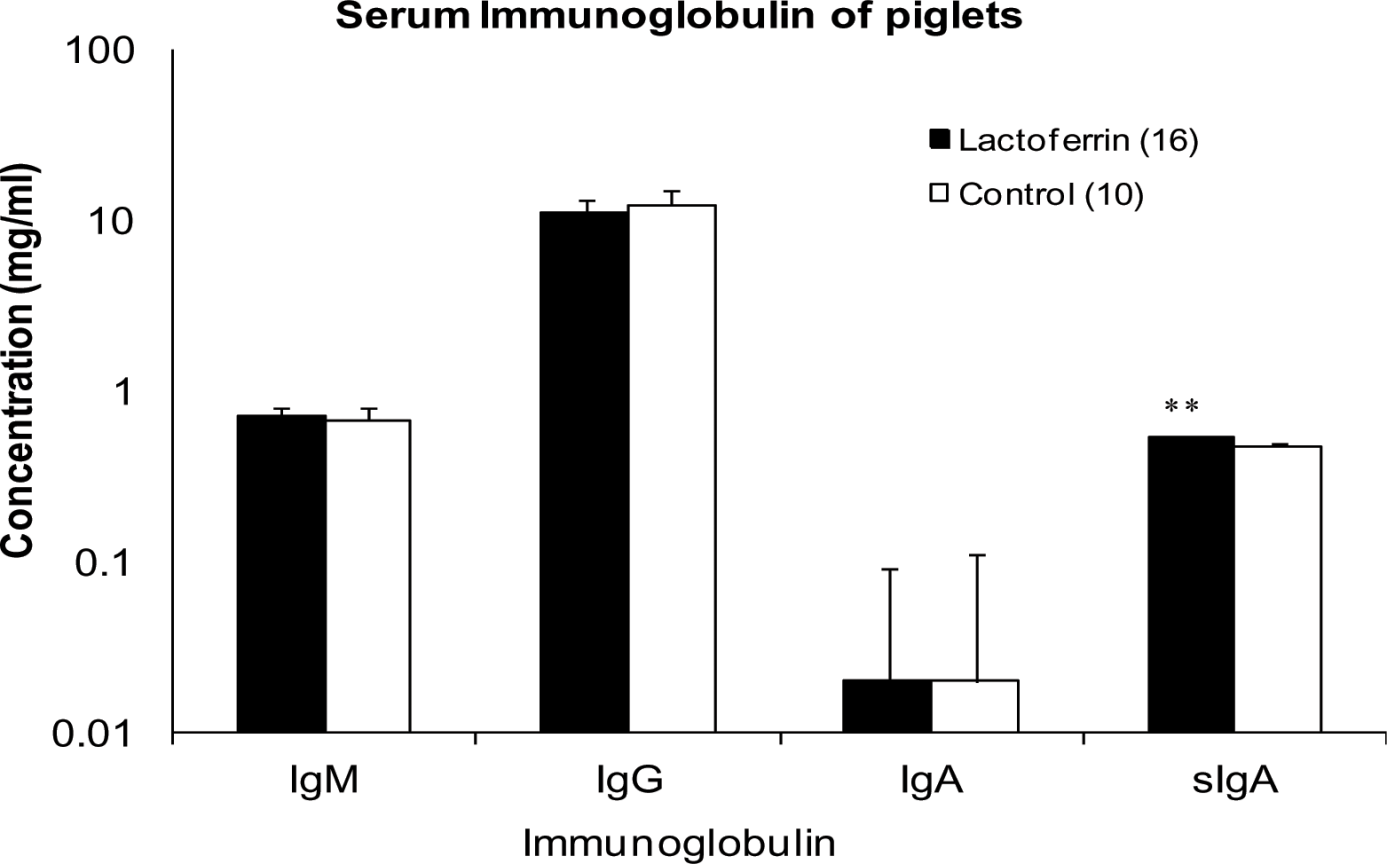
The effect of maternal lactoferrin supplementation (1g/day) during gestation and lactation (~135 days) on serum immunoglobulin concentrations in piglets. Concentration of Immunoglobulin IgM, IgG, IgA and sIgA in the serum sample of piglets collected at weaning. *p<0.001. Values are mean ± SEM.

Mean concentrations of IgM, IgG, IgA and sIgA in the serum of piglets from the treatment and control groups at weaning are shown in Fig 7. A significantly higher concentration of sIgA was detected in the serum of piglets from the LF group (0.54 ±0.01 mg/ml) compared to the piglets from the control group (0.48 ± 0.01 mg/ml; p = 0.001). There was no significant difference between the two groups in the concentrations of IgM, IgG, IgA (p> 0.05).

## Discussion

Maternal bLF supplementation is a beneficial nutritional intervention which increases hemoglobin and total serum iron in pregnant women [32] and reverses some of the IUGR-induced sequelae, including brain hippocampal changes [33]. In this study, we demonstrated that maternal oral administration of LF to gilts during gestation and lactation did not increase gestational weight and the birth weight of piglets, but significantly increased milk production throughout 4 stages of lactation which resulted in an increased live weight gain of piglets to weaning. The mechanism of LF supplementation on the physiological effects of gilt milk production is not well understood. However, the total milk production capacity of the mammary gland is determined by the mammary epithelial cell population and its biosynthetic capacity [34, 35]. In an earlier study Hagiwara, Shinoda [36] found that LF stimulates the epithelial cell growth in rat intestinal cell line. Thus, increased milk production in LF treatment gilts resulting in increased growth of their piglets implies that LF may stimulate proliferation and differentiation of the mammary epithelial cell population and enhances the central component of secretory mammary gland growth in first pregnancy mammals. The fact that the mammary epithelial cell population may not be fully developed in gilts, where LF may contribute to mammary gland maturation.

The significant increase in milk production in LF treated gilts may also be due to the increased litter size, with an additional piglet available to provide a greater suckling intensity [37, 38]. This may have influenced the initiation of lactation on day 1 prior to the rationalisation of litter size on day 2 in the current study. Suckling intensity is a major determinant of both mammary development and milk yield of sows. The effect of suckling intensity on potential milk yield and piglet growth has been extensively investigated [37–40]. In pigs, there are several sow-litter interactions including suckling intensity, litter size, suckling frequency and age and size of piglets that influence the potential milk yield of the sow. Milk production is the key factor in determining the rate of piglet growth and development. Milk removal from the mammary gland is of critical importance in sustaining milk secretion [38]. Treatment piglets tended to exhibit an increased mean birth weight and a significant weaning weight (1.41 kg and 5.34 kg respectively) compared to control piglets (1.24 kg and 4.45 kg respectively). Heavier piglets are able to stimulate increased milk flow and may empty out mammary glands completely during feeding, unlike lighter weight piglets. Increased stimulation leads to greater oxytocin release and greater milk let-down and therefore increased piglet live weight gain [38, 39]. Milk yield peaks were found at day nine of lactation in sows [38]. In the present study, we estimated milk production at 4 time points during 19 days of lactation. The LF group displayed a higher milk production compared with the control group gilts throughout the lactation. In particular, on lactation day 7, the LF group gilts produced more than double the volume of milk than that of the control group gilts (LF: 629.76 g; Control: 207g).

Part of the growth response to maternal LF supplementation may be the result of the immunomodulatory effects of LF in significantly increasing the concentration of serum sIgA levels in their piglets. Yang et al reported [7] increased expression of mRNA for some non- specific immune factors like the antimicrobial peptide 39- residue proline-arginine- rich peptide (PR-39) and protegrin-1 in LF supplemented piglets, resulting in improved intestinal morphology post weaning. Therefore, maternal LF supplementation from postnatal day one may improve gut mucosal immunity resulting in a decreased incidence of *E*.*coli* infection [41–43]. This can potentially improve piglet feed intake, with a consequent increase in live weight gain during lactation. The significant increase in piglet live weight gain matches the increased milk availability associated with maternal LF supplementation. Marshall, Hurley [38] demonstrated that milk production is a significant limiting factor in the growth and development potential of piglets. The increased supply of functionally important minor proteins and immunoglobulins in milk most likely provided increased immunoprotection and antimicrobial factors to the neonate [44]. Furthermore, piglet birth live weights affect mortality rates and future growth and development (33). This was attributed to a lower colostrum intake, which subsequently compromised health and increased mortality rates in the first week post farrowing [45]. The more vigorous suckling piglets in litters from LF treated gilts may have consumed more colostrum, which would have assisted in developing mucosal immunity relative to piglets from the control group and therefore improved their growth potential.

Mammalian milk is rich in lipids, carbohydrates, proteins and non-nutritional products, which are of functional significance to the growth physiology of the developing offspring [39, 46]. Among the non-nutritional products immunoglobulin A, LF, macrophages and lysosomes help in protecting the digestive tract against potential pathogens [46]. LF exhibited an immunomodulatory effect on the gilts and their piglets as documented by a significant increase in the concentration of IgA in gilt serum and sIgA in piglet serum. It was documented from the earlier studies that dietary LF supplementation to weaning piglets enhanced serum concentration of IgA, IgG and IgM [41, 47]. In the current study, we demonstrated if maternal LF supplementation to gilts during pregnancy and lactation can produce a similar immune response in gilts themselves, as well as in piglets. Various immunomodulatory effects of orally administered LF have been documented in a variety of mammalian species. Debabbi et al [48] reported increased concentrations of IgA and IgG in intestinal fluids of mice after oral LF administration. Butler [49] reported that pig serum IgA levels ranged from 1.32–1.56 mg/ml, which is consistent with the concentrations recorded in LF treated (2.11 ± 0.33 mg/ml) and control gilts (0.8 ± 0.45 mg/ml). The significant response in LF supplemented gilts may be due to LF’s positive charge which facilitated its binding to the surface of various cells within the host immune system. This in turn has been shown to trigger signalling pathways that can regulate cellular responses, including differentiation, activation and proliferation of immune cells [50]. The significant increase in serum sIgA in piglets on day 19 of lactation with LF treatment in the present study may have been due to increased proliferation of B- lymphocytes in the gut [42, 43, 51].

Despite the significant difference found in gilt serum IgA and piglet sIgA concentration in the LF treatment group compared with the control, no significant differences in immunoglobulin concentration for gilt serum IgM, IgG, sIgA and piglet IgM, IgG and IgA were detected between two groups. sIgA, a subclass of IgA, which is the predominant immunoglobulin in mucosal surfaces produced by B-lymphocytes adjacent to the mucosal cells. These are transported to the interior of these lymphocytes and released into their secretions. The level of faecal sIgA antibody is correlated with higher virus neutralizing capacity and increased viral clearance [52]. The high sIgA in piglet serum implies that maternal LF supplementation plays a key role in protecting vulnerable areas such as the oral cavity, lungs, and gut of its offspring from invading pathogens. However the limitation of this study is that we were not able to determine any anti- or pro-inflammatory cytokines in the piglets, due to the limited volume of blood samples were allowed to be collected from the piglets at the commercial pig farm. Future studies should be carried out to confirm the role of LF on anti- or pro-inflammatory cytokines in pregnant gilts and sows [53].

## Conclusion

First pregnancy mammals contribute a large proportion of progeny to any growing commercial herd. LF supplementation during pregnancy and lactation in gilts significantly improved milk production and increased serum IgA levels in gilt, as well as piglets’ growth and serum sIgA concentration. There is, therefore, potential for the use of LF as a functional ingredient in the feed of pregnant gilts and sows to boost the health status and productivity of their litters.

## Supporting information

S1 Table Breed information of the experimental gilts of treatment and control group. (DOCX)

S2 Table Total no of gilts from each breed line for the lactoferrin and control group. (DOCX)

S3 Table Nutritional composition of commercial pig feed. (DOCX)

S4 Table Information on the litter size of the farrowing gilts of lactoferrin and control group before and after cross-fostering. (DOCX)
