# Supplementary material for: Dietary lactoferrin supplementation to gilts during gestation and lactation improves pig production and immunity
Source: PLoS One. 2017 Oct 12;12(10):e0185817. doi: 10.1371/journal.pone.0185817 (PMC5638254; doi:10.1371/journal.pone.0185817)
Supplement: S1 Table — (DOCX) [file pone.0185817.s001.docx]

**S1 Table.** **Breed information of the experimental gilts of treatment and control group.**

| **Treatment**  **Group** | **Gilt ID** | **Breed line** | **Treatment**  **Group** | **Gilt ID** | **Breed line** |
| --- | --- | --- | --- | --- | --- |
| LF | 32909 | 3 | Control | 32823 | 3 |
|  | 32950 | 3 |  | 32831 | 3 |
|  | 32962 | 3 |  | 32970 | 3 |
|  | 32901 | 3 |  | 32829 | 3 |
|  | 32981 | 3 |  | 32820 | 3 |
|  | 32982 | 3 |  | 10731 | 3 |
|  | 32991 | 3 |  | B10932 | 3 |
|  | 32803 | 3 |  | 32842 | 3 |
|  | 32809 | 3 |  | 32801 | 3 |
|  | 32827 | 3 |  | 32838 | 3 |
|  | 32989 | 3 |  | 32814 | 3 |
|  | 32826 | 3 |  | 32996 | 3 |
|  | B10805 | 3 |  | 32813 | 3 |
|  | B10915 | 3 |  | B10536 | 3 |
|  | 32834 | 3 |  | 32825 | 3 |
|  | B10991 | 3 |  | B10763 | 3 |
|  | 32966 | 3 |  | B10876 | 3 |
|  | B10940 | 3 |  | Y07385 | 2 |
|  | 21562 | 2 |  | 21501 | 2 |
|  | 21554 | 2 |  | 21557 | 2 |
|  | 21568 | 2 |  | Y07399 | 2 |
|  | 40777 | 4 |  | R03908 | 4 |
|  | 40791 | 4 |  | R03975 | 4 |
|  | R03826 (40794) | 4 |  | 40795 | 4 |
|  | 70755 | 7 |  | 40781 | 4 |
|  | 70753 | 7 |  | 70757 | 7 |
|  | 91314 | 9 |  | 91304 | 9 |
|  | 91301 | 9 |  | 91300 | 9 |
|  | 91310 | 9 |  | 91316 | 9 |
|  | 91308 | 9 |  | W06643 | 9 |
