# Supplementary material for: Dietary lactoferrin supplementation to gilts during gestation and lactation improves pig production and immunity
Source: PLoS One. 2017 Oct 12;12(10):e0185817. doi: 10.1371/journal.pone.0185817 (PMC5638254; doi:10.1371/journal.pone.0185817)
Supplement: S2 Table — (DOCX) [file pone.0185817.s002.docx]

S2 Table. Total no of gilts from each breed line for the lactoferrin and control group.

| **Treatment**  **Group** | **Breed line 2** | **Breed line 3** | **Breed line 4** | **Breed line 7** | **Breed line 9** |
| --- | --- | --- | --- | --- | --- |
| LF | 3 | 19 | 3 | 2 | 3 |
| Control | 4 | 17 | 4 | 1 | 4 |
