# Supplementary material for: Dietary lactoferrin supplementation to gilts during gestation and lactation improves pig production and immunity
Source: PLoS One. 2017 Oct 12;12(10):e0185817. doi: 10.1371/journal.pone.0185817 (PMC5638254; doi:10.1371/journal.pone.0185817)
Supplement: S3 Table — (DOCX) [file pone.0185817.s003.docx]

S3 Table. Nutritional composition of commercial pig feed.

| Nutrients | Unit | Product | Product |
| --- | --- | --- | --- |
|  |  | Lactation sow | Dry sow |
| Pig DE | MJ/kg | 14.05 | 13.022 |
| Pig ME | Mcal/kg | 3.225 | 2.989 |
| Moisture | % | 9.187 | 9.608 |
| Crude protein | % | 17.938 | 14.3 |
| Lysine | % | 1.12 | 0.819 |
| Methionine | % | 0.32 | 0.264 |
| Methionine + Cystine | % | 0.706 | 0.601 |
| Threonine | % | 0.709 | 0.553 |
| Isoleucine | % | 0.78 | 0.586 |
| Leucine | % | 1.273 | 0.954 |
| Tryptophan | % | 0.22 | 0.167 |
| Arginine | % | 1.185 | 0.924 |
| Histidine | % | 0.453 | 0.354 |
| Valine | % | 0.862 | 0.71 |
| Available Lysine | % | 0.936 | 0.651 |
| Crude fat | % | 5.487 | 5.5 |
| Crude fibre | % | 4.787 | 6.285 |
| Ash | % | 6.006 | 6.846 |
| Calcium | % | 0.89 | 1.149 |
| Available phosphorus | % | 0.4 | 0.4 |
| Total phosphorus | % | 0.829 | 0.909 |
| Salt | % | 0.342 | 0.387 |
| Sodium | % | 0.127 | 0.16 |
| Chloride | % | 0.213 | 0.235 |
| Choline | mg/kg | 1909.948 | 1598.472 |
