# Supplementary material for: Dietary lactoferrin supplementation to gilts during gestation and lactation improves pig production and immunity
Source: PLoS One. 2017 Oct 12;12(10):e0185817. doi: 10.1371/journal.pone.0185817 (PMC5638254; doi:10.1371/journal.pone.0185817)
Supplement: S4 Table — (DOCX) [file pone.0185817.s004.docx]

**S4Table. Information on the litter size of the farrowing gilts of lactoferrin and control group before and after cross-fostering.**

| **Lactoferrin gilts** | | | | | |  |  |  |  |  |
| --- | --- | --- | --- | --- | --- | --- | --- | --- | --- | --- |
| Gilt number | ID | Day 1 litter size | Litter size after cross fostering | Mean piglet BW at weaning after cross fostering | | No of weaned treatment piglets |  |  |  |  |
| 1 | 32991 | 16 | Gilt moved before weaning |  | |  |  |  |  |  |
| 2 | 32909 | 13 | 11 | 4.18 | | 9 |  |  |  |  |
| 3 | 32981 | 12 | 11 | 4.87 | | 10 |  |  |  |  |
| 4 | 32803 | 16 | 10 | 4.67 | | 8 |  |  |  |  |
| 5 | 40777 | 8 | 10 | 6.40 | | 8 |  |  |  |  |
| 6 | 32982 | 12 | Gilt moved before weaning |  | |  |  |  |  |  |
| 7 | 21562 | 13 | 11 | 6.16 | | 11 |  |  |  |  |
| 8 | 70755 | 6 | 6 | 10.70 | | 6 |  |  |  |  |
| 9 | R03826 | 11 | 9 | 5.93 | | 9 |  |  |  |  |
| 10 | 32826 | 11 | 9 | 4.67 | | 6 |  |  |  |  |
| 11 | 91314 | 6 | Gilt moved before weaning |  | |  |  |  |  |  |
| 12 | 91310 | 10 | 9 | 4.28 | | 7 |  |  |  |  |
| 13 | 32989 | 12 | 11 | 4.15 | | 3 |  |  |  |  |
| 14 | 40791 | 9 | Gilt moved before weaning |  | |  |  |  |  |  |
| 15 | B10805 | 16 | 8 | 4.89 | | 8 |  |  |  |  |
| 16 | B10915 | 11 | 7 | 4.29 | | 6 |  |  |  |  |
| 17 | B10991 | 11 | 11 | 5.12 | | 4 |  |  |  |  |
| 18 | 21568 | 16 | 9 | 3.65 | | 9 |  |  |  |  |
| 19 | 32966 | 2 | 10 | 6.52 | | 2 |  |  |  |  |
| 20 | B10940 | 11 | 8 | 4.51 | | 8 |  |  |  |  |
| 21 | 32901 | 12 | Gilt moved before weaning |  | |  |  |  |  |  |
| **Control gilts** | | | | | |  |  |  | **Casein control gilts** | **Casein control gilts** |
| No. gilt | ID | Day 1 litter size | Cross fostering litter size | | Mean piglets BW at weaning after cross fostering | No of weaned piglets staying within treatment groups |  |  |  |  |
| 1 | 32831 | 11 | 9 | | 4.77 | 6 |  |  |  |  |
| 2 | 32970 | 11 | 11 | | 4.97 | 11 |  |  |  |  |
| 3 | 32820 | 10 | 6 | | 4.13 | 6 |  |  |  |  |
| 4 | 91304 | 2 | Gilt moved before weaning | |  |  |  |  |  |  |
| 5 | B10731 | 10 | Gilt moved before weaning | |  |  |  |  |  |  |
| 6 | 32842 | 13 | 10 | | 3.55 | 4 |  |  |  |  |
| 7 | B10932 | 4 | Gilt moved before weaning | |  |  |  |  |  |  |
| 8 | 91300 | 10 | Gilt moved before weaning | |  |  |  |  |  |  |
| 9 | 32814 | 13 | 6 | | 4.53 | 6 |  |  |  |  |
| 10 | 32838 | 12 | 11 | | 5.99 | 10 |  |  |  |  |
| 11 | Y07385 | 11 | 10 | | 3.77 | 10 |  |  |  |  |
| 12 | 32813 | 11 | 10 | | 4.29 | 6 |  |  |  |  |
| 13 | B10536 | 15 | Gilt moved before weaning | |  |  |  |  |  |  |
| 14 | 21560 | 7 | 10 | | 5.39 | 4 |  |  |  |  |
| 15 | R03975 | 11 | 9 | | 5.03 | 9 |  |  |  |  |
| 16 | B10876 | 15 | 8 | | 3.87 | 8 |  |  |  |  |
| 17 | B10763 | 10 | Gilt moved before weaning | |  |  |  |  |  |  |
| 18 | 70757 | 8 | Gilt moved before weaning | |  |  |  |  |  |  |
